# Beyond the Liver: A Comprehensive Review of Strategies to Prevent Hepatocellular Carcinoma

**DOI:** 10.3390/jcm13226770

**Published:** 2024-11-11

**Authors:** Natchaya Polpichai, Sakditad Saowapa, Pojsakorn Danpanichkul, Shu-Yen Chan, Leandro Sierra, Johanna Blagoie, Chitchai Rattananukrom, Pimsiri Sripongpun, Apichat Kaewdech

**Affiliations:** 1 Department of Medicine, Weiss Memorial Hospital, Chicago, IL 60640, USA; npolpichai@weisshospital.com (N.P.); schan@weisshospital.com (S.-Y.C.); jblagoie@weisshospital.com (J.B.); 2 Department of Medicine, Texas Tech University Health Science Center, Lubbock, TX 79430, USA; sakditad.saowapa@ttuhsc.edu (S.S.); pdanpani@ttuhsc.edu (P.D.); 3 Department of Medicine, Cleveland Clinic Foundation, Cleveland, OH 44195, USA; sierral@ccf.org; 4 Division of Gastroenterology and Hepatology, Department of Medicine, Faculty of Medicine, Srinagarind Hospital, Khon Kaen University, Khon Kaen 40002, Thailand; chitra@kku.ac.th; 5 Gastroenterology and Hepatology Unit, Division of Internal Medicine, Faculty of Medicine, Prince of Songkla University, Songkhla 90110, Thailand; spimsiri@medicine.psu.ac.th

**Keywords:** hepatocellular carcinoma (HCC), liver cancer, prevention, surveillance

## Abstract

Background/Objectives: Hepatocellular carcinoma (HCC) is a leading cause of cancer-related mortality worldwide, primarily developing in the context of chronic liver disease. Traditional prevention has focused on liver-specific interventions like antiviral therapies and surveillance. However, extrahepatic factors also significantly contribute to HCC risk. This review explores comprehensive strategies for HCC prevention, including both hepatic and extrahepatic factors. Methods: An extensive literature search of peer-reviewed articles up to October 2024 was conducted, focusing on studies addressing HCC prevention strategies. Studies that focused on both hepatic and extrahepatic factors were included. Data were extracted and synthesized to provide an overview of current prevention strategies and their effectiveness in reducing HCC incidence. Results: Hepatitis B vaccination and antiviral treatments for hepatitis B and C significantly reduce HCC incidence. Lifestyle modifications—such as reducing alcohol consumption, maintaining a healthy weight through diet and exercise, and smoking cessation—are crucial in lowering HCC risk. Environmental measures to limit exposure to aflatoxins and other hazards also contribute to prevention. Regular surveillance of high-risk groups enables early detection and improves survival rates. Emerging strategies like immunotherapy and gene therapy show potential for further reducing HCC risk. Conclusions: A comprehensive approach combining medical interventions, lifestyle changes, and environmental controls is essential for effectively decreasing HCC incidence globally. Implementing these combined measures could significantly reduce the global burden of HCC.

## 1. Introduction

Hepatocellular carcinoma (HCC), the most common primary liver cancer, represents a significant global health challenge due to its high morbidity and mortality rates. As the third leading cause of cancer-related deaths worldwide, HCC accounted for an estimated 529,202 new cases and 483,875 deaths in 2021, according to the Global Burden of Disease Study, 2021 [1]. The global burden of HCC continues to rise, driven by the rising prevalence of chronic liver diseases and metabolic disorders.

The incidence and mortality rates of HCC vary geographically, with the highest occurrences reported in East Asia and sub-Saharan Africa [2]. This variation is largely attributed to the prevalence of chronic hepatitis B virus (HBV) infection in these regions [3]. Chronic HBV infection remains a predominant etiological factor for HCC, responsible for approximately 50% of cases worldwide [4]. In contrast, the incidence of HCC in Western countries has been rising due to factors such as chronic hepatitis C virus (HCV) infection, alcohol-associated liver disease (ALD), and metabolic dysfunction-associated steatotic liver disease (MASLD), formerly known as nonalcoholic fatty liver disease (NAFLD) [5,6]. HBV contributes to hepatocarcinogenesis through direct viral effects, such as integration into the host genome, and indirect effects like chronic inflammation leading to cirrhosis. Similarly, chronic HCV infection increases HCC risk by inducing chronic liver inflammation and fibrosis, eventually progressing to cirrhosis [7].

A recent epidemiological study in the Asia–Pacific region showed an increase in HCC cases caused by steatotic liver disease (SLD), including both ALD and MASLD. This finding aligns with global trends, where the increased prevalence of metabolic syndrome, alcohol consumption, and SLD is often attributed to the widespread adoption of Westernized diets in many countries [8]. Similar to Western countries, these proportions are expected to shift over the next 2–3 decades, with a decline in HCV-related HCC cases and an increase in those linked to metabolic syndrome [9].

ALD remains a significant contributor to HCC development, particularly in regions with high alcohol consumption. Prolonged excessive alcohol intake leads to liver cirrhosis, creating a conducive environment for the malignant transformation of hepatocytes [10]. The emergence of MASLD and its progressive form, metabolic dysfunction-associated steatohepatitis (MASH), has become a growing concern. These conditions are associated with metabolic syndrome components such as obesity, type 2 diabetes, dyslipidemia, and hypertension [11]. MASLD is now recognized as one of the leading causes of chronic liver disease worldwide and is increasingly associated with HCC development, even in the absence of cirrhosis [12]. In addition, obesity and diabetes not only contribute to MASLD but also independently elevate the risk of HCC through mechanisms involving insulin resistance and chronic inflammation [13]. Moreover, autoimmune liver disease, though a less common cause of HCC, has been associated with a lower risk of HCC compared to chronic viral hepatitis, potentially due to differences in the regulatory T cell background [14].

Exposure to aflatoxins, toxic metabolites produced by *Aspergillus* species contaminating food supplies, is another significant risk factor for HCC, especially in developing countries with inadequate food storage facilities. Aflatoxin B₁ has been implicated in inducing mutations in the p53 tumor suppressor gene, thereby promoting hepatocarcinogenesis [15]. Genetic and metabolic disorders, including hereditary hemochromatosis, alpha-1 antitrypsin deficiency, and Wilson’s disease, also predispose individuals to chronic liver injury and subsequent HCC development [16]. Environmental and lifestyle factors also contribute to HCC risk; for example, tobacco smoking has been associated with an elevated risk of liver cancer due to the presence of carcinogens that can induce DNA damage [17].

Understanding the multifactorial etiology of HCC is essential for developing effective prevention strategies. This comprehensive review aims to explore current approaches to preventing HCC by addressing these diverse risk factors. By focusing on primary prevention through vaccination, antiviral therapies, lifestyle modifications, and environmental interventions, as well as secondary prevention via surveillance and early detection in high-risk populations, we aim to contribute to the reduction in HCC incidence and mortality worldwide.

## 2. Primary Prevention Strategies

### 2.1. Vaccination Programs

#### Hepatitis B Virus Vaccination

Vaccination against HBV is one of the most effective primary prevention measures for HCC. Universal HBV vaccination programs have significantly reduced the prevalence of HBV infection and the incidence of HBV-related HCC, particularly in endemic regions [18]. A landmark study in Taiwan demonstrated that the incidence of HCC in children decreased substantially following the implementation of a nationwide HBV vaccination program [19]. The World Health Organization (WHO) recommends that all infants receive the HBV vaccine within 24 h of birth, followed by at least two additional doses, to decrease mother-to-child transmission (MTCT) globally [20]. Therefore, HBV vaccination remains an essential component of global health strategies aimed at reducing the burden of HCC. Additionally, infants born to hepatitis B surface antigen-positive mothers should receive hepatitis B immunoglobulin (HBIG) within 12–24 h of birth [21].

### 2.2. Hepatitis C Virus Vaccine Development

Currently, there is no approved vaccine for hepatitis C virus (HCV) due to the virus’s high genetic variability and the complex immune responses required for protection [22]. However, ongoing research aims to develop an effective HCV vaccine by inducing a broad spectrum of neutralizing antibodies and a strong cellular immune response to overcome these challenges. Such a vaccine could significantly reduce new infections and, consequently, HCV-related hepatocellular carcinoma (HCC) cases by preventing chronic infection and subsequent liver disease progression [23]. Recently, a phase 1–2 randomized, double-blind, placebo-controlled trial evaluated an HCV vaccine regimen using a recombinant chimpanzee adenovirus 3 vector, followed by a booster with modified vaccinia Ankara, in adults at risk for HCV infection. While the vaccine was safe, induced HCV-specific T-cell responses, and reduced peak HCV RNA levels, it did not significantly prevent chronic HCV infection compared to placebo [24].

## 3. Antiviral Therapies

### 3.1. Treatment of Chronic HBV Infection

Antiviral therapies for chronic HBV infection aim to suppress viral replication, reduce liver inflammation, and prevent progression to cirrhosis and HCC. Two classes of approved antiviral agents, including interferon (IFN) and nucleos(t)ide analogs (NA), can efficiently control HBV infection [25]. However, clinical relapse is frequent after treatment [26,27,28]. Nucleos(t)ide analogs—such as entecavir (ETV), tenofovir disoproxil fumarate (TDF), and tenofovir alafenamide (TAF)—have demonstrated high efficacy in inhibiting HBV DNA polymerase, leading to viral suppression and thereby reducing hepatic inflammation and the progression to cirrhosis and HCC [29]. Long-term antiviral therapy has been associated with a significant reduction in HCC risk among patients with chronic HBV infection [30]. Notably, the risk of HCC differs among the various NAs. Several studies suggest that TDF users have a lower incidence of HCC compared to ETV users as demonstrated in Table 1. However, the observational design of these studies warrants cautious interpretation. In addition, various prospective studies and meta-analyses of IFN therapy have shown a 34–41% lower incidence of HCC compared with untreated patients [31].

### 3.2. Treatment of Chronic HCV Infection

The introduction of direct-acting antivirals (DAAs) has revolutionized the treatment of chronic HCV infection, achieving sustained virologic response (SVR) rates exceeding 95% across all genotypes, which effectively equates to a virological cure [37]. Achieving SVR has been associated with a significant reduction in the risk of HCC development, as it reduces hepatic inflammation and fibrosis progression [38]. Studies have demonstrated that patients who achieve SVR have a substantially lower incidence of HCC compared to those with active infection [39]. A decrease in liver stiffness, which reflects improved liver fibrosis, has been associated with a reduced risk of HCC. A study by Facciorusso et al. provides valuable insights into the long-term effects of antiviral therapy on liver stiffness. In patients with chronic HCV infection who achieved an SVR, liver stiffness showed a substantial reduction, decreasing from 12.3 kPa at baseline to 6.6 kPa over a five-year follow-up period. Notably, the decline in liver stiffness was most pronounced during the first year post-treatment, followed by a slower, sustained improvement in subsequent years [40]. Early observational studies reported an increased incidence of HCC, along with a higher rate of recurrence in cirrhotic patients who achieved SVR following DAA treatment [41,42]. These findings raised concerns that the rapid suppression of viral replication by DAAs could disrupt immune surveillance, potentially increasing the likelihood of HCC development [41,42]. However, recent meta-analysis suggested that there is no evidence of a differential risk for HCC occurrence or recurrence following SVR between DAA and interferon (IFN)-based therapies [43].

## 4. Lifestyle Modifications

### 4.1. Alcohol Consumption Reduction

Chronic alcohol intake is a well-established risk factor for HCC, as it leads to liver cirrhosis and promotes carcinogenesis through mechanisms such as oxidative stress, inflammation, and impaired immune surveillance [44]. Studies have demonstrated that decreasing alcohol consumption lowers the incidence of HCC, particularly in populations with high rates of ALD [45]. Currently, there is no longer a so-called ‘safe amount’ of alcohol intake [46]. A large database study included 28 million individuals globally indicated that the level of alcohol consumption that minimizes harm from alcohol is zero [47]. Similarly, a 4.9-year follow-up study of 58,927 Korean MASLD patients reported that light and moderate drinkers were also associated with worsening fibrosis scores [48]. Another study also supported alcohol abstinence, particularly in patients who had metabolic syndrome [49]. Public health interventions aimed at reducing alcohol use—including taxation, regulation of availability, and educational campaigns—have been effective in diminishing alcohol-related harm and, consequently, the risk of HCC.

### 4.2. Weight Management and Physical Activity

Obesity and metabolic syndrome are associated with MASLD, which can progress to metabolic dysfunction-associated steatohepatitis (MASH) and subsequently increase the risk of HCC [50]. Engaging in regular physical activity and maintaining a healthy body weight have been shown to reduce hepatic fat accumulation, improve insulin sensitivity, and decrease liver inflammation, thereby lowering the risk of HCC development [51,52]. In patients with MASLD, a weight loss of at least 7–10% is recommended to achieve significant improvements in steatosis and fibrosis [53]. Furthermore, a meta-analysis found that physical activity reduces liver cancer risk and mortality in a dose-dependent manner [54]. Engaging in at least two hours of physical activity per week is necessary to reduce liver cancer mortality.

### 4.3. Dietary Interventions

Dietary interventions play a significant role in the primary prevention of HCC by modulating risk factors such as obesity, metabolic syndrome, and MASLD, as well as directly influencing liver carcinogenesis through bioactive compounds [55]. Hypercaloric diets high in trans and saturated fats, cholesterol, and fructose-sweetened beverages increase visceral adiposity and promote hepatic lipid accumulation, leading to MASLD [56]. Consuming a diet rich in fruits, vegetables, and whole grains—as emphasized in the Mediterranean diet—reduces the risk of HCC due to the antioxidant and anti-inflammatory properties of these foods, which reducing oxidative stress and inflammation, key processes in hepatic carcinogenesis [57]. Limiting the intake of red and processed meats is recommended, as they contain heme iron and carcinogenic compounds formed during processing and high-temperature cooking—such as heterocyclic amines and polycyclic aromatic hydrocarbons—that have been linked to increased HCC risk [58].

Regular consumption of coffee and green tea has been inversely associated with HCC incidence as demonstrated in Table 2, possibly due to their antioxidant compounds like chlorogenic acids, caffeine, and catechins such as epigallocatechin gallate (EGCG), which exhibit anti-inflammatory and anti-carcinogenic properties [59,60]. Omega-3 fatty acids, found in fatty fish and certain plant oils, have demonstrated protective effects against HCC development by modulating liver fat metabolism, reducing hepatic steatosis, and exerting anti-inflammatory and anti-fibrotic effects [61].

### 4.4. Smoking Cessation

Tobacco smoking is an established independent risk factor for HCC, with meta-analyses demonstrating that smokers have a significantly higher risk of developing HCC compared to non-smokers [17]. This risk is further amplified in patients with MASLD [68]. Carcinogenic compounds in tobacco smoke contribute to hepatocarcinogenesis through mechanisms such as oxidative stress induction, DNA damage, and promotion of hepatic inflammation and fibrosis [69]. Notably, individuals who quit smoking more than 30 years ago exhibit an HCC risk nearly equivalent to that of never-smokers [70].

## 5. Environmental Control

### 5.1. Reducing Aflatoxin Exposure

Aflatoxin B₁, produced by *Aspergillus* species and primarily transmitted through contaminated food, is a significant risk factor for HCC in developing countries [15]. Strategies to reduce aflatoxin exposure—such as improving agricultural practices, ensuring proper food storage, and utilizing processing methods to prevent fungal contamination—have been effective in decreasing HCC incidence. Implementing regulations to monitor and control aflatoxin levels in food supplies is also critical for reducing exposure in affected populations [71].

### 5.2. Occupational and Environmental Hazard Management

Exposure to industrial chemicals such as vinyl chloride, arsenic, and polychlorinated biphenyls (PCBs) has been associated with an increased risk of HCC development [72,73]. Occupational settings involving the handling of these carcinogenic substances require stringent safety protocols—including the use of personal protective equipment and proper ventilation systems—to minimize exposure. Environmental measures, such as regulating industrial emissions and preventing contamination of water and soil, are also essential in reducing the prevalence of HCC linked to environmental toxins.

### 5.3. Management of Genetic and Metabolic Disorders

Genetic disorders such as hereditary hemochromatosis, alpha-1 antitrypsin deficiency and Wilson’s disease can lead to chronic liver injury and increase HCC risk [74]. Early diagnosis through genetic screening and appropriate management, including phlebotomy for hemochromatosis, can prevent disease progression [75,76].

## 6. Pharmacological Interventions

### 6.1. Aspirin

Aspirin demonstrates its chemopreventive effects primarily through the inhibition of cyclooxygenase-2 (COX-2), which diminishes the synthesis of pro-inflammatory prostaglandins and reduces chronic liver inflammation, a key driver of hepatocarcinogenesis [77]. Additionally, its antiplatelet properties disrupt platelet–tumor cell interactions that facilitate tumor growth and metastasis [78]. Several studies and recent meta-analyses have demonstrated that regular aspirin use is associated with a significant reduction in HCC risk as demonstrated in Table 3, particularly among individuals with chronic liver disease [79,80,81,82,83,84,85,86,87,88,89,90,91]. However, long-term aspirin therapy may increase the risk of gastrointestinal bleeding and hemorrhagic stroke, necessitating a careful risk–benefit assessment [92].

### 6.2. Statin

As hydroxymethylglutaryl-CoA (HMG-CoA) reductase inhibitors, statins lower cholesterol levels and exhibit anti-inflammatory and immunomodulatory effects that may help inhibit carcinogenesis. They also induce apoptosis and inhibit the proliferation of hepatic cancer cells by modulating the mevalonate pathway and suppressing oncogenic signaling pathways, such as Ras/Raf/MEK/ERK [93]. Numerous studies have consistently demonstrated that statin users with chronic liver disease have a lower risk of developing HCC compared to non-users as demonstrated in Table 4. However, despite these benefits, statin use can lead to myopathy or, in severe cases, rhabdomyolysis, particularly in patients with decompensated cirrhosis, who may receive low-dose statins and require intensive monitoring. A recent study suggests that the protective effects of statins against HCC may be primarily associated with lipophilic statins, such as simvastatin and atorvastatin, while hydrophilic statins, including pravastatin and rosuvastatin, showed no significant benefit [94]. Similarly, a recent meta-analysis found that lipophilic statins had a greater chemopreventive effect on HCC risk compared to hydrophilic statins, with an adjusted odds ratio of 0.51 (95% CI: 0.46–0.57) versus 0.77 (95% CI: 0.58–1.02) [95].

### 6.3. Metformin

Metformin, a widely used antidiabetic medication, has shown potential in reducing HCC incidence through multiple mechanisms. It activates AMP-activated protein kinase (AMPK), which inhibits the mammalian target of rapamycin (mTOR) pathway, thereby suppressing tumor cell growth and proliferation [104]. Metformin also lowers insulin and insulin-like growth factor (IGF) levels, which are implicated in hepatocarcinogenesis [105]. Clinical evidence from studies indicates that diabetic patients treated with metformin have a significantly reduced risk of developing HCC as demonstrated in Table 5. However, metformin use may be associated with lactic acidosis, particularly in patients with renal impairment, necessitating careful patient selection [106].

### 6.4. Glucagon-like Peptide-1 (GLP-1) Agonists

**Figure 1:**
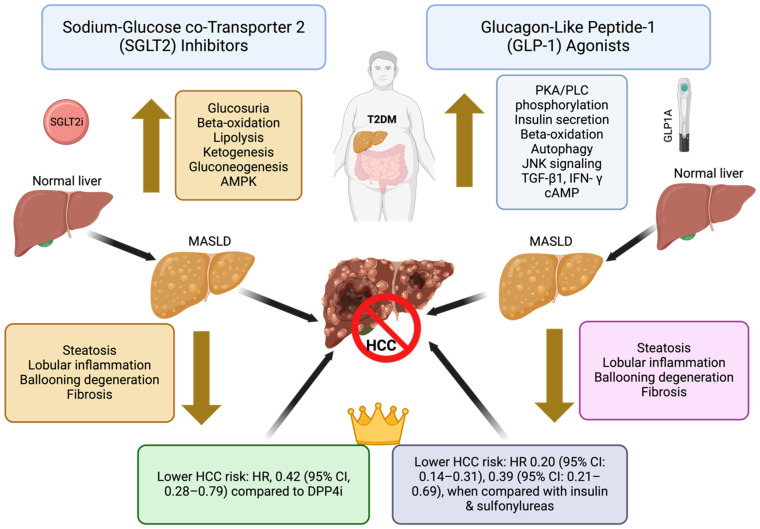
Summary of the mechanisms of action of SGLT2 inhibitors and GLP-1 analogs for HCC prevention. AMPK—5′-adenosine monophosphate-activated protein kinase; cAMP—cyclic adenosine monophosphate; GLP-1—glucagon-like peptide-1; HCC—hepatocellular carcinoma; IFN-γ—interferon gamma; JNK—c-Jun N-terminal kinase; MASLD—metabolic dysfunction-associated steatotic liver disease; PKA—protein kinase A; PLC—phospholipase C; SGLT2i—sodium-glucose cotransporter 2 inhibitor; TGF-β1—transforming growth factor beta 1.

GLP-1 agonists have emerged as potential chemopreventive agents for HCC, particularly in patients with type 2 diabetes. These agents enhance insulin sensitivity, reduce hyperinsulinemia, and exhibit anti-inflammatory properties, collectively contributing to a lower risk of hepatocarcinogenesis [113]. Additionally, GLP-1 agonists induce apoptosis in hepatic cancer cells through the modulation of the PI3K/Akt pathway [113]. Several studies have reported a significant reduction in HCC incidence among GLP-1 agonist users [114]. A recent large cohort study by Wang et al. demonstrated that GLP-1 agonists are associated with a lower risk of incident HCC in patients with type 2 diabetes, with hazard ratios of 0.20 (95% CI: 0.14–0.31), 0.39 (95% CI: 0.21–0.69), and 0.63 (95% CI: 0.26–1.50) when compared with insulin, sulfonylureas, and metformin, respectively [114]. However, potential adverse effects, including gastrointestinal disturbances and an increased risk of pancreatitis, warrant careful consideration and monitoring. Figure 1 summarizes the mechanism of action of GLP-1 agonists in HCC prevention.

### 6.5. Sodium-Glucose Co-Transporter 2 (SGLT2) Inhibitors

SGLT2 inhibitors, primarily used for managing type 2 diabetes (T2DM), improve glycemic control and reduce insulin resistance, both of which are implicated in the development of HCC, as shown in the mechanisms summarized in Figure 1. These agents also exert anti-inflammatory and anti-fibrotic effects, helping to reduce liver damage, particularly in patients with MASLD [115]. Emerging studies suggest that SGLT2 inhibitors may lower the risk of HCC by improving non-invasive markers of steatosis and even fibrosis in patients with T2DM [116]. Recently, a large retrospective cohort study from Hong Kong compared the risk of new-onset HCC in patients with T2DM treated with SGLT2 inhibitors versus dipeptidyl peptidase-4 inhibitors (DPP4i). After propensity score matching and adjustments, SGLT2-inhibitor use was associated with a significantly lower risk of HCC (HR, 0.42; 95% CI, 0.28–0.79) compared to DPP4i, with this protective effect also observed in patients with cirrhosis, advanced fibrosis, HBV, and HCV infections. The findings were consistent across different risk models and sensitivity analyses [117].

### 6.6. Thiazolidinediones

Thiazolidinediones, such as pioglitazone, are insulin sensitizers used in T2DM management and have been studied for their role in reducing HCC risk. These drugs activate peroxisome proliferator-activated receptor gamma (PPARγ), which enhances insulin sensitivity, reduces liver steatosis, and has anti-inflammatory effects, which may contribute to a reduced risk of HCC [118]. Some observational studies suggest that pioglitazone use is associated with a reduced incidence of HCC, particularly in diabetic patients [119]. A recent meta-analysis by Arvind et al. demonstrated that thiazolidinedione use is associated with a reduced risk of HCC in patients with type 2 diabetes (aOR = 0.92, 95% CI: 0.86–0.97; I^2^ = 43%) [119]. However, concerns about the potential risk of bladder cancer and cardiovascular complications with long-term pioglitazone use warrant careful consideration [120].

### 6.7. Angiotensin-Converting Enzyme (ACE) Inhibitors and Angiotensin Receptor Blockers (ARBs)

ACE inhibitors and ARBs have anti-fibrotic and anti-inflammatory properties, which can reduce liver fibrosis, a major precursor to HCC [121]. One meta-analysis suggested that patients using ACE inhibitors or ARBs have a lower risk of developing HCC [122].

### 6.8. Vitamin D Supplements

Vitamin D has anti-inflammatory, immunomodulatory, and anti-proliferative effects, which may reduce the risk of HCC [123]. A meta-analysis conducted by Yi et al. demonstrated that vitamin D deficiency is significantly associated with an increased risk of liver cancer, with a pooled relative risk (RR) of 2.16 (95% CI: 1.20–3.88; *p* = 0.01), and supplementation could theoretically lower this risk by modulating pathways involved in cell proliferation and inflammation [124,125]. However, results from vitamin D supplementation trials on liver outcomes are limited.

### 6.9. Nutraceuticals and Herbal Supplements

Nutraceuticals and herbal supplements have garnered attention for their potential role in preventing HCC due to their antioxidant, anti-inflammatory, and anti-proliferative properties. Compounds such as curcumin, found in turmeric, and resveratrol, found in grapes, have shown promise in preclinical studies by inhibiting tumor growth, reducing oxidative stress, and modulating inflammatory pathways linked to liver [126,127]. Silymarin, derived from milk thistle, has also demonstrated hepatoprotective effects by reducing liver fibrosis and inflammation, thereby potentially lowering the risk of HCC [128]. Despite its general reputation for hepatoprotective properties, curcumin has been linked to documented cases of drug-induced liver injury (DILI) [129]. Additionally, herbal supplements can interact with conventional medications and vary in quality and potency, underscoring the importance of regulation and standardization.

## 7. Secondary Prevention Strategies

### Target Populations for Surveillance [130]

Surveillance for HCC is recommended for individuals at high risk of developing the disease [131]. According to international guidelines, the following groups should undergo regular surveillance: patients with cirrhosis of any etiology except for those with Child–Pugh class C cirrhosis who are not on the waiting list for liver transplantation; individuals with chronic HBV infection, even without cirrhosis, in certain subgroups, including males aged at least 40 years, females aged at least 50 years, and patients with a family history of HCC at any age; and patients with chronic HCV infection with advanced fibrosis (Metavir stage F3) in the absence of cirrhosis. Information about the incidence of HCC in patients with non-viral chronic liver diseases without cirrhosis—such as alcohol-associated steatohepatitis and MASH, autoimmune liver disease, genetic haemochromatosis, alpha-1 antitrypsin deficiency, and Wilson’s disease—is limited [29]. The recommended surveillance strategy is liver ultrasound every six months, with or without the measurement of serum alpha-fetoprotein (AFP), to facilitate early detection and improve survival outcomes [29]. Studies have demonstrated that surveillance leads to earlier detection of HCC, increased eligibility for curative treatments, and reduction in mortality compared to those who did not undergo surveillance [132,133].

The strategy of HCC surveillance was based on a randomized controlled trial from China involving 18,816 CHB patients [133]. In the study, the patients were divided into two groups, a surveillance group (9373 patients) and a control (9443 patients) group; patients in the surveillance group received ultrasonography of the liver and AFP at six-month intervals. HCC-related mortality was significantly lower in the screened group (83.2 per 100,000) than in the control group (131.5 per 100,000), with a mortality rate ratio of 0.63 (95% CI 0.41–0.98), indicating that surveillance every six months reduced HCC mortality by 37%. Currently, according to international practice guidelines, surveillance for HCC should be undertaken in the high-risk group of patients, particularly in cirrhosis, as up to 90% of HCC occurs in cirrhotic patients [74,134,135,136]. Recently, a meta-analysis of over 93,000 HCC patients showed that approximately one-third of MASLD-related HCC occurred in patients without cirrhosis [137]. Of note, the proportion of MASLD-related HCC undergoing HCC surveillance was lower than that of HCC from other causes, and the tumors were usually detected at a larger size. These findings highlight the necessity of HCC surveillance among MASLD patients, especially in the absence of cirrhosis [137].

## 8. Screening Modalities

### 8.1. Imaging Techniques

Ultrasound (US): Ultrasound is the most widely used screening tool due to its non-invasive nature, availability, and cost-effectiveness. It is recommended every six months for high-risk patients [138]. Increasingly, a higher proportion of HCC cases are being diagnosed through structured surveillance programs, particularly those adhering to a semi-annual screening schedule. Regular use of ultrasound in these programs enables the early detection of HCC, allowing for timely intervention and improved patient outcomes [139].

Contrast-enhanced ultrasound (CEUS): CEUS enhances lesion characterization by providing superior visualization of tumor vascularity [140]. International guidelines recommend CEUS when conventional ultrasound results are inconclusive due to its higher sensitivity and specificity [74]. However, its application is limited by availability and the requirement for specialized training.

Computed tomography (CT): CT is a widely used imaging modality for the diagnosis and staging of HCC. Multi-phase contrast-enhanced CT, particularly with arterial and portal venous phases, enables the visualization of the characteristic vascular patterns of HCC [141]. HCC lesions typically exhibit hyperenhancement during the arterial phase and washout during the portal venous or delayed phases, which are critical imaging features for diagnosis [141]. Despite its advantages, the use of CT for routine HCC screening is limited due to concerns about radiation exposure and its relatively lower sensitivity in detecting small lesions (<1 cm) compared to MRI [134].

Magnetic resonance imaging (MRI): MRI, particularly with hepatobiliary contrast agents like gadolinium ethoxybenzyl diethylenetriamine pentaacetic acid (Gd-EOB-DTPA), is considered superior to CT for HCC screening, particularly in detecting early-stage and small lesions [142]. This modality is particularly advantageous in cases lacking the typical vascular pattern, as it enables the identification of small HCC lesions through the characteristic double hypo-intensity in the portal-venous and hepatobiliary phases. This distinctive feature provides a significant diagnostic advantage for early-stage detection, thereby improving patient outcomes [143]. MRI provides better soft tissue contrast and can distinguish between benign and malignant lesions more accurately than CT. In addition to conventional sequences, diffusion-weighted imaging (DWI) enhances the ability to detect and characterize small tumors, and dynamic contrast-enhanced imaging identifies the vascular characteristics of HCC, similar to CT [144]. Although MRI is generally more sensitive and specific for early HCC, its higher cost and limited availability in some regions remain challenges in routine screening programs.

### 8.2. Biomarkers

Alpha-fetoprotein (AFP): AFP is the most studied serum biomarker for HCC surveillance. However, its sensitivity and specificity are suboptimal when used alone, as elevated levels can be seen in other liver diseases [145]. Combining AFP measurement with ultrasound improves detection rates [146].

Des-gamma-carboxy prothrombin (DCP) and AFP-L3: These biomarkers have shown promise in detecting HCC but are not widely adopted due to limited availability and higher costs [147].

Emerging biomarkers: Research is ongoing to identify novel biomarkers, such as circulating tumor DNA, microRNAs, and proteins, to improve early detection [148].

## 9. Risk Stratification Models

Risk stratification models identify individuals at the highest risk of developing HCC, allowing for more personalized surveillance strategies. These models integrate demographic, clinical, and laboratory variables to accurately predict HCC risk. By utilizing risk scores, healthcare providers can tailor the intensity of surveillance, with higher risk patients receiving more frequent or advanced imaging modalities to enhance early detection and improve outcomes.

### Examples of Risk Scores

- REACH-B score: Developed for HBV-infected patients without cirrhosis, incorporating age, gender, ALT levels, HBeAg status, and HBV DNA levels [149].
- PAGE-B score: Used for HBV patients on antiviral therapy, including age, gender, and platelet count [149].
- aMAP score: Applicable to both HBV and HCV patients, considering age, male gender, albumin-bilirubin grade, and platelet count [150,151].
- GALAD score: Combines gender, age, AFP-L3, AFP, and DCP to enhance predictive accuracy for HCC in diverse populations [152].
- GAAD score: Incorporates gender, age, AFP, and DCP levels to improve HCC risk prediction, particularly in at-risk populations such as those with chronic liver disease [153].

## 10. Conclusions

**Figure 2:**
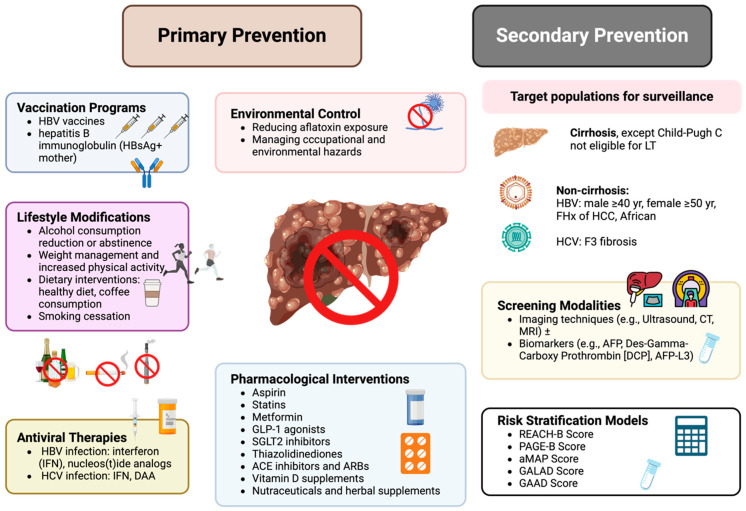
Summary of overall strategies for HCC prevention. HBV—hepatitis B virus; HCV—hepatitis C virus; HCC—hepatocellular carcinoma; IFN—interferon; DAA—direct-acting antiviral; GLP-1—glucagon-like peptide-1; SGLT2—sodium-glucose cotransporter 2; ACE—angiotensin-converting enzyme; ARB—angiotensin II receptor blocker; AFP—alpha-fetoprotein; DCP—des-gamma-carboxy prothrombin; REACH-B—risk estimation for hepatocellular carcinoma in chronic hepatitis B; PAGE-B—platelet, age, gender-HBV score; aMAP—age, male gender, albumin-bilirubin score, and platelet count; GALAD—gender, age, AFP, AFP-L3, and DCP; GAAD—Gender, Age, AFP, and AFP-L3.

HCC continues to represent a significant global health burden due to its high incidence and mortality rates. This comprehensive review highlights that effective prevention strategies are diverse, including primary measures such as vaccination, antiviral therapies, lifestyle modifications, and environmental control, alongside secondary prevention strategies like regular surveillance in high-risk populations, as summarized in Figure 2. Pharmacological interventions, including aspirin, statins, and metformin, offer promising chemopreventive benefits. Moving forward, it is imperative to enhance global efforts to expand access to preventive measures, invest in public health education, and foster research into novel preventive therapies. A coordinated, worldwide approach is essential to further reduce the incidence and improve outcomes in HCC.

## Figures and Tables

**Table 1:** Incidence of hepatocellular carcinoma in TDF users compared to ETV users.

Authors, Year	Population	Key Findings	Limitation
Zhang, 2019 [32]	TDF: 1574 ETV: 2124	The incidence of HCC was significantly lower among the TNF group than the ETV group [risk ratio (RR), 95% confidence interval (CI) of 0.66 (0.49, 0.89), *p* = 0.008].	No TAF
Choi, 2021 [33]	TDF: 16,101 ETV: 45,686	TDF treatment was associated with a significantly lower risk of HCC than entecavir (hazard ratio (HR), 0.80; 95% CI, 0.69–0.93; *p* = 0.003; I^2^ = 13%).	No TAF
Cheung, 2020 [34]	ETV: 56,346 TDF: 28,662	TDF was associated with a lower HCC risk (adjusted HR (aHR): 0.81; 95% CI: 0.67–0.99).	No TAF
Dave, 2021 [35]	ETV: 49,606 TDF: 19,730	The risk of HCC was not statistically different between ETV and TDF (incidence rate ratio (IRR), 1.28; 95% CI, 0.99–1.66).	No TAF
Tan, 2022 [36]	TDF: 10,534 ETV: 13,735	Tenofovir was associated with decreased HCC incidence compared with entecavir (stratified Cox HR, 0.85 [95% CI, 0.76–0.94] at 5 years; *p* = 0.002).	No TAF

**Table 2:** Incidence of hepatocellular carcinoma in regular coffee and green tea consumers compared to non-drinkers.

Authors, Year	Study Design	Population	Key Findings
Yu, 2023 [62]	Meta-analysis	Twenty-one studies involving 2,492,625 participants and 5980 cases of HCC reported coffee intake, 18 studies involving 1,481,647 participants and 6985 cases of HCC reported green tea intake, and seven studies reported both coffee intake and green tea intake.	The results showed that a higher coffee (RR = 0.53; 95% CI: 0.47–0.59; I^2^ = 0.0%; *p* heterogeneity = 0.634) or green tea (RR = 0.80; 95% CI: 0.67–0.95; I^2^ = 72.30%; *p* heterogeneity < 0.001) intake may be associated with a lower risk of HCC.
Kenedy, 2017 [63]	Meta-analysis	Eighteen cohorts, involving 2,272,642 participants	An extra two cups per day of coffee was associated with a 35% reduction in the risk of HCC (RR 0.65, 95% CI 0.59 to 0.72).
Tamura, 2018 [64]	Cohort	Coffee consumption: 23,327 Nondrinkers: 7497	The incidence of HCC in relation to coffee consumption were 0.65 (95% CI: 0.46–0.93) for less than once per day, 0.63 (95% CI: 0.39–1.02) for once per day, and 0.40 (95% CI: 0.20–0.79) for twice per day or more, compared with nondrinkers.
Setiawan, 2015 [65]	Cohort	<1 cup per day: 31,056 1 cup per day: 45,717 2–3 cups per day: 32,593 ≥4 cups per day: 8218 Nondrinkers: 44,438	High levels of coffee consumption were associated with a reduced risk of incident HCC. Compared with non-coffee drinkers, those who drank 2–3 cups per day had a 38% reduction in HCC risk (RR = 0.62; 95% CI: 0.46–0.84), while those who drank ≥4 cups per day had a 41% reduction in HCC risk (RR = 0.59; 95% CI: 0.35–0.99).
Lai, 2013 [66]	Cohort	Coffee consumption: 26,370 Nondrinkers: 667	Coffee intake was inversely associated with incident liver cancer (RR per cup per day = 0.82, 95% CI: 0.73–0.93).
Gelatti, 2005 [67]	Case-Control	Coffee consumption: 26,370 Nondrinkers: 647	Coffee consumption was associated with a decreasing risk of HCC with a clear dose-effect relation. With respect to non-drinking subjects, the odds ratios (ORs) were: 0.8, (95% CI 0.4–1.3) for 1–2 cups/day, 0.4 (95% CI 0.2–0.8) for 3–4 cups/day and 0.3 (95% CI 0.1–0.7) for 5 or more cups/day.

**Table 3:** Incidence of hepatocellular carcinoma in aspirin users compared to non-users.

Authors, Year	Study Design	Population	Key Findings
Bian, 2024 [91]	Meta-analysis	A total of 13 articles were included	The incidence of HCC in aspirin users was lower than that in non-aspirin users (HR 0.75; 95% CI, 0.68–0.83; *p* < 0.001; I^2^ = 90.0%).
Wang, 2023 [84]	Meta-analysis	Nineteen studies including three prospective and 16 retrospective studies	Aspirin use had a 30% lower risk of HCC, HR = 0.70, 95% CI 0.63–0.76, I^2^ = 84.7%, *p* < 0.001).
Ma, 2023 [90]	Meta-analysis	Twenty five studies of HCC incidence	Aspirin use correlated with lower incidence of HCC (HR: 0.75, 95% CI: 0.71–0.80).
Zeng, 2023 [85]	Meta-analysis	Pooled analysis of 11 studies with 2,190,285 patients	Aspirin use was associated with reduced HCC risk overall (HR: 0.48; 95% CI: 0.27–0.87).
Tan, 2023 [86]	Meta-analysis	Six observational studies with 71,211 patients	There was a 54% reduction in the incidence of HCC among regular aspirin users (HR (95% CI): 0.46(0.31–0.67), *p* < 0.001).
Wang, 2022 [87]	Meta-analysis	Eighteen studies (16 cohort, 2 case-control) were included	Aspirin users were less likely to develop HCC than non-users (aOR = 0.54; 95% CI: 0.44–0.66).
Zhou, 2022 [88]	Meta-analysis	A total of sixteen studies (2,781,100 participants) were included.	There was lower incidence of HCC in aspirin users than that in non-aspirin users (HR, 0.56; 95% CI, 0.46–0.69; *p* < 0.001).
Liao, 2020 [89]	Cohort	Aspirin-treated HCV carriers: 2980 Non-aspirin-treated HCV carriers: 7771	HCC incidence in the aspirin users was significantly lower than that in the non-aspirin users (aHR = 0.56, 95% CI = 0.43–0.72, *p* < 0.001).
Simon, 2020 [83]	Cohort	Aspirin users: 14,205 Non-users: 36,070	The estimated cumulative incidence of HCC was 4.0% among aspirin users and 8.3% among non-users of aspirin in patients with chronic HBV and HCV (aHR 0.69; 95% CI, 0.62 to 0.76).
Lee, 2019 [80]	Cohort	Daily aspirin for >90 days: 2123 Never received aspirin: 8492	Aspirin therapy was associated with a reduced HCC risk in patients with chronic HBV (HR, 0.71; 95% CI, 0.58–0.86; *p* < 0.001).

**Table 4:** Incidence of hepatocellular carcinoma in statin users compared to non-users.

Authors, Year	Study Design	Population	Key Findings
Rafsanjani, 2024 [96]	Meta-analysis	Forty studies were included with a total sample of 5,732,948 participants, including 68,698 HCC cases.	Statin use was associated with a 44% lower risk of HCC compared to non-use (RR 0.56, 95% CI 0.50–0.63, *p* < 0.001).
Zeng, 2023 [85]	Meta-analysis	A pooled analysis of 10 studies with 1,774,476 patients	Statin use was associated with reduced HCC risk overall (HR: 0.52; 95% CI: 0.37–0.72).
Zhang, 2023 [97]	Meta-analysis	Five studies involving 684,363 patients were included.	The results suggested a significantly lower risk of HCC among statin users with MASLD [OR = 0.59; 95% CI 0.39–0.89; I^2^ = 87.90%].
Wang, 2022 [95]	Meta-analysis	Thirty-two studies reporting 56,838 cases of hepatocellular carcinoma in 4,963,518 persons were included.	Statin users were less likely to develop HCC than non-users (aOR, 0.58; 95% CI: 0.51–0.67).
Tsai, 2024 [98]	Cohort	Statin users: 500 Non-statin users: 106	Statin use in patients with chronic HCV who failed antiviral therapy was associated with a lower risk of HCC compared to non-users (adjusted sub-distribution hazard ratio (aSHR) = 0.50; *p* < 0.001).
Vell, 2023 [99]	Cohort	Statin users: 56,109 Non-statin users: 148,948	Statin users demonstrated a 42% lower hazard ratio for the development of HCC (HR, 0.58; 95% CI, 0.35–0.96; *p* = 0.04).
Sinn, 2023 [100]	Cohort	Statin users: 2915 Non-statin users: 8525	Patients with chronic HBV in the statin user group had a higher incidence of HCC compared to the non-user group, with an aHR of 0.56 (95% CI: 0.39–0.80).
Zou, 2023 [101]	Cohort	Statin users: 73,385 Non-statin users: 199,046	Statin users with MASLD had a 53% lower risk of developing HCC compared with non-users (HR 0.47; 95% CI: 0.36–0.60).
Kraglund, 2023 [102]	Cohort	Statin users: 1438 Non-statin users: 118,460	Using statins continuously for 5 years compared with not using statins in patients with ALD cirrhosis resulted in an RR of HCC of 0.67 (95% CI: 0.45–0.91).
Pinyopornpanish, 2021 [103]	Cohort	Statin users: 440 Non-statin users: 632	Statin use in patients with MASH-related advanced liver fibrosis (bridging fibrosis [F3] and cirrhosis [F4]) was associated with a lower risk of developing HCC (HR, 0.40, 95% CI, 0.24–0.67, *p* = 0.001).

**Table 5:** Incidence of hepatocellular carcinoma in metformin users compared to non-users.

Authors, Year	Study Design	Population	Key Findings
Li, 2022 [107]	Meta-analysis	The study included 9 case-control studies (including 248,433 participants) and 15 cohort studies (including 1,203,832 participants)	Metformin use was associated with a decreased risk of HCC in patients with diabetes (OR/RR = 0.59, 95% CI 0.51–0.68, I^2^ = 96.5%, *p* < 0.001).
Zhang, 2013 [108]	Meta-analysis	A total of seven studies were included, including three cohort studies and four case-control studies	The analysis showed a reduced risk of HCC in metformin users versus non-users in diabetic patients (RR 0.24, 95% CI 0.13–0.46, *p* < 0.001).
Tsai, 2023 [109]	Cohort	Non-diabetes: 6468 Metformin users: 647 Non-metformin users: 134	The 5-year cumulative HCC incidence was 10.9% in non-metformin users and 2.6% in metformin users, compared to 3.0% in individuals without DM (aHR 2.83; 95% CI 1.57–5.08 and aHR 1.46; 95% CI 0.98–2.19, respectively).
Tseng, 2018 [110]	Cohort	Metformin users: 2642 Non-metformin users: 619	The HRs of HCC development for the first (<25.7 months), second (25.7–56.9 months) and third (>56.9 months) tertiles of cumulative duration of metformin therapy were 0.89 (0.81–0.98), 0.50 (0.46–0.56) and 0.23 (0.21–0.26), respectively.
Chen, 2015 [111]	Cohort	Metformin users: 4774 Non-metformin users: 53,037	Metformin use in chronic HBV patients was associated with a reduction in the incidence of liver cancer (aHR of 0.97, 95% CI, 0.84–1.14).
Lee, 2011 [112]	Cohort	Metformin users: 11,390 Non-metformin users: 4327	Metformin use in patients with diabetes was significantly associated with a lower risk of developing HCC (HR 0.06, 95% CI, (0.02–0.16)).

## Data Availability

Data supporting the findings of this study are available from the corresponding author upon request.
